
JOURNAL INFORMATION
==============================
NLM Title Abbreviation: ScientificWorldJournal
Journal ID: ScientificWorldJournal
NLM Journal ID: 101131163
Journal ID: TSWJ

The Scientific World Journal

ISSN: 2356-6140
EISSN: 1537-744X
Publisher: Wiley

ARTICLE INFORMATION
==============================
PMCID: PMC5823181
PMID: 19347230
DOI: 10.1100/tsw.2009.26
Article ID: 891856
Article version: 1
Subjects: Clinical Images

Abdominal Cocoon: A Case of Sclerosing Encapsulating Peritonitis

Turagam Mohit *
Are C.
Velagapudi P.
Holley J.
University of Illinois, Urbana-Champaign, IL, USA
*Mohit Turagam: turagam2@illinois.eduAcademic Editor: Daniel Batlle

Publication date: 2009
Electronic publication date: 2009 Mar 31
Volume: 9
First page: 201
Last page: 203
Received 2009 Feb 11; Revised 2009 Feb 16; Accepted 2009 Feb 16
Copyright: Copyright © 2009 Mohit Turagam et al.
Copyright year: 2009
License: This is an open access article distributed under the Creative Commons Attribution License, which permits unrestricted use, distribution, and reproduction in any medium, provided the original work is properly cited.
License URL: https://creativecommons.org/licenses/by/3.0/

Keywords: sclerosing peritonitis encapsulated, peritoneal dialysis, peritoneal sclerosis

==============================
Clinical Image
*TheScientificWorldJOURNAL* (2009) 9, 201–203
ISSN 1537-744X; DOI 10.1100/tsw.2009.26

### Abdominal Cocoon: A Case of Sclerosing Encapsulating Peritonitis

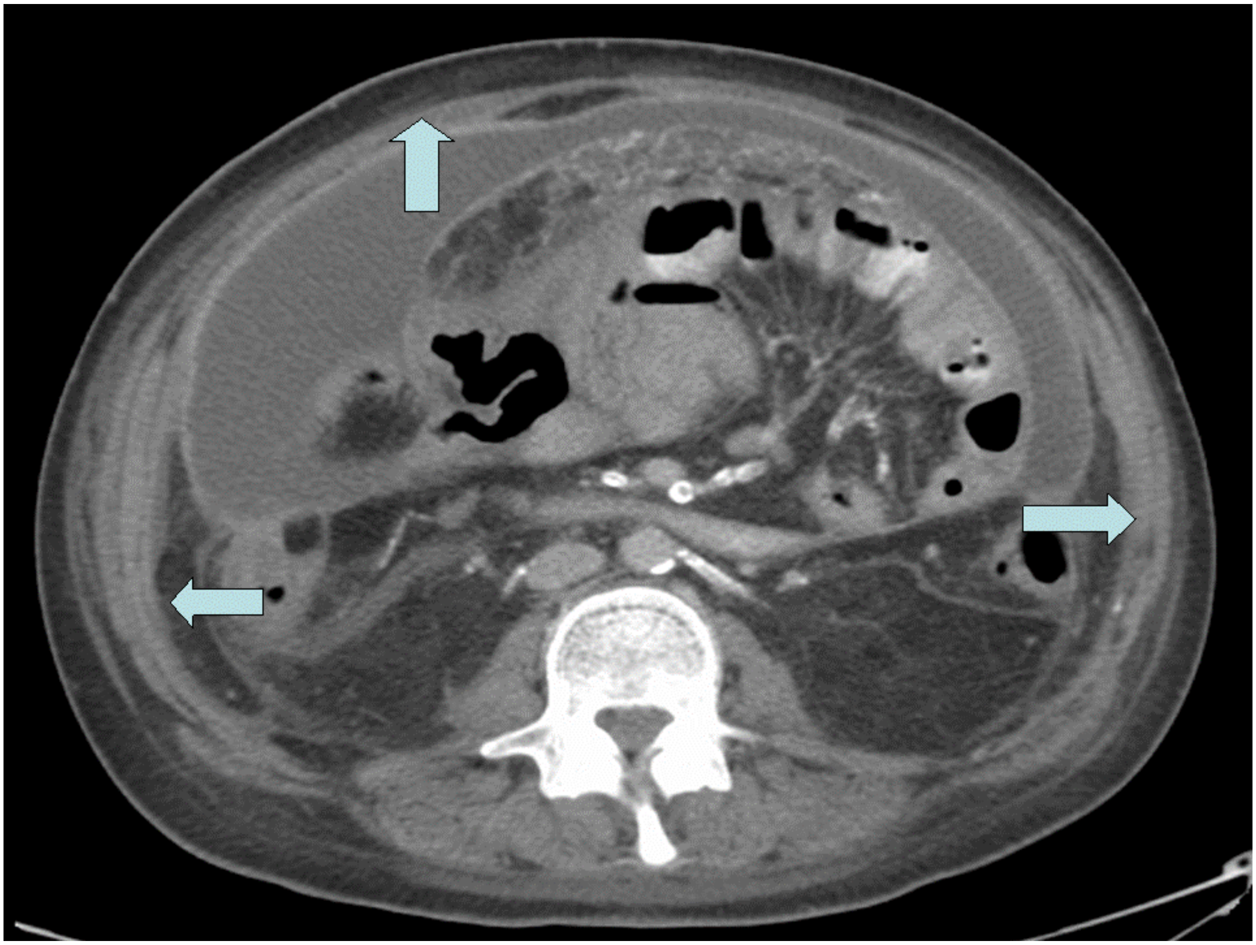

**FIGURE**. CT image of the abdomen shows dilated small bowel loops surrounded by thickened fibrous peritoneum.

M. Turagam*, C. Are, P. Velagapudi, and J. Holley
*University of Illinois, Urbana-Champaign*

**E-mail**: turagam2@illinois.edu

*Received February 11, 2009; Revised February 16, 2009; Accepted February 16, 2009; Published March 31, 2009*

**KEYWORDS:** sclerosing peritonitis encapsulated, peritoneal dialysis, peritoneal sclerosis

*Corresponding author.
©2009 with author.
Published by TheScientificWorld; www.thescientificworld.com

### INTRODUCTION

Sclerosing peritonitis encapsulatum (SPE) is also known as encapsulating peritoneal sclerosis or abdominal cocoon. SPE is a rare, but lethal, complication of continuous ambulatory peritoneal dialysis (CAPD) and represents an advanced stage of peritoneal sclerosis.

### CASE

A 43-year-old male with Type 1 diabetes and end-stage renal disease (ESRD) was admitted with persistent vomiting and diffuse abdominal pain for 1 week. Prior to this admission, he had been hospitalized three times in a span of 2 months due to recurrent peritonitis. He had been on CAPD for 8 years with a recent change to hemodialysis (HD) 2 weeks prior. Examination revealed soft abdomen with diminished bowel sounds, diffuse tenderness, and no rigidity or guarding. Labs revealed a white cell count of 13,000/µl, hemoglobin 10.7 g/dl, blood urea nitrogen 40 mg/dl, creatinine 5.3 mg/dl, AST 61 IU/l, ALT 44 IU/l, alkaline phosphatase of 1336 IU/l, and normal total bilirubin. The computerized tomography (CT) scan of the abdomen showed presence of ascites, peritoneal thickening, cholelithiasis, and possible cholecystitis. Ascitic fluid analysis and cultures were negative for infection. The patient underwent open cholecystectomy, but there was no improvement in the severity of abdominal pain. A repeat CT scan 1 week later showed reaccumulation of ascites, omental thickening, and peritoneal enhancement findings consistent with SPE. The patient was started on total parenteral nutrition (TPN), but failed to improve and died in the hospital 2 weeks later.

### DISCUSSION

Etiology of SPE is unknown, but the pathogenesis due to persistent exposure of mesothelial cells to transforming growth factor β and proliferation of peritoneal fibroblasts is widely accepted. Predisposing factors include recurrent peritonitis and chronic peritoneal irritation due to dialysate. The annual incidence of SPE in CAPD patients is 0.37 per 1000 patient years, with a male predominance (5:2), and the median duration on CAPD is 62 months. Presentation is generally nonspecific with abdominal pain, distension, nausea, and vomiting. Patients often experience a reduced peritoneal clearance or impaired peritoneal ultrafiltration. Classic CT scan findings include thickened adhered bowel loops, loculated ascites, and peritoneal enhancement. The definitive diagnosis is by laparotomy and biopsy. Initial management involves the removal of dialysis catheter, discontinuation of CAPD, initiation of TPN, and bowel rest. The use of steroids, immunosuppressives, and tamoxifen has been reported to be beneficial, but needs to be validated. Surgery is high risk for fistula formation and anastomotic leakage. Despite various therapeutic modalities, the mortality rate is 56–93%.

### CONCLUSION

Patients who are and were on CAPD presenting with symptoms and signs of bowel obstruction and otherwise unexplained abdominal pain should be evaluated for SPE.

### REFERENCES

1. Foo, K.T., Ng, K.C., Rauff, A., Foong, W.C., and Sinniah, R. (1978) Unusual small intestinal obstruction in adolescent girls: the abdominal cocoon. *Br. J. Surg*. **65,** 427–430.
2. Holland, P. (1990) Sclerosing encapsulating peritonitis in chronic ambulatory peritoneal dialysis. *Clin. Radiol*. **41,** 19–23.

3. Afthentopoulos, I.E., Passadakis, P., Oreopoulos, D.G., and Bargman, J. (1998) Sclerosing peritonitis in continuous ambulatory peritoneal dialysis patients: one center's experience and review of the literature. *Adv. Ren. Replace. Ther.* **5(3),** 157–167.
4. Rigby, R.J. and Hawley, C.M. (1998) Sclerosing peritonitis: the experience in Australia. *Nephrol. Dial. Tranplant*. **13,** 154–159.
5. Izumotani, T., Ishimura, E., Yamamoto, T., Otoshi, T., Okuno, S., Inaba, M., Kim, M., and Nishizawa, Y. (2001) Correlation between peritoneal mesothelial cell cytology and peritoneal histopathology with respect to prognosis in patients on continuous ambulatory peritoneal dialysis. *Nephron* **89(1),** 43–49.
6. Kawanishi, H., Kawaguchi, Y., Fukui, H., Hara, S., Imada, A., Kubo, H., Kin, M., Nakamoto, M., Ohira, S., and Shoji, T. (2004) Encapsulating peritoneal sclerosis in Japan: a prospective, controlled, multicenter study. *Am. J. Kidney Dis*. **44,** 729–737.
7. Nomoto, Y., Kawaguchi, Y., Kubo, H., Hirano, H., Sakai, S., and Kurokawa, K. (1996) Sclerosing encapsulating peritonitis in patients undergoing continuous ambulatory peritoneal dialysis: a report of the Japanese Sclerosing Encapsulating Peritonitis Study Group. *Am. J. Kidney Dis*. **28,** 420–427.
8. Nakamoto, H., Kawaguchi, Y., and Suzuki, H. (2002) Encapsulating peritoneal sclerosis in patients undergoing continuous ambulatory peritoneal dialysis in Japan. *Adv. Perit. Dial.* **18,** 119–123.
9. Chin, A.I. and Yeun, J.Y. (2006) Encapsulating peritoneal sclerosis: an unpredictable and devastating complication of peritoneal dialysis. *Am. J. Kidney Dis*. **47(4),** 697–712.

**This article should be cited as follows:**

Turagam, M., Are, C., Velagapudi, P., and Holley, J. (2009) Abdominal cocoon: a case of sclerosing encapsulating peritonitis. *TheScientificWorldJOURNAL* **9**, 201–203. DOI 10.1100/tsw.2009.26.
